# Genetic and Immunohistochemical Profiling of Malignant Mesothelioma With Brain Metastasis: A Report of Two Cases

**DOI:** 10.1002/cnr2.70571

**Published:** 2026-05-08

**Authors:** Paul M. Harary, Yusuke S. Hori, Ruchit Jain, Ahed H. Kattaa, Melanie Hayden Gephart, Michael F. Gensheimer, Scott G. Soltys, David J. Park, Steven D. Chang

**Affiliations:** ^1^ Department of Neurosurgery Stanford University School of Medicine Stanford California USA; ^2^ Department of Radiation Oncology Stanford University School of Medicine Stanford California USA

**Keywords:** brain metastases, genetics, malignant mesothelioma, stereotactic radiosurgery, whole‐brain radiotherapy

## Abstract

**Background:**

Brain metastasis (BM) occurs in < 3% of malignant mesothelioma (MM) cases and is associated with an aggressive disease course. While genomic profiling has provided insight into molecular alterations in MM, the characteristics of MM with brain involvement remain unexplored. Data are particularly limited for MM of pericardial origin, an exceedingly rare tumor which comprises < 1% of mesotheliomas.

**Cases:**

We describe the clinical course and genetic profiles of two patients with BM from MM, both of whom exhibited atypical presentations, including neurological symptoms, diagnosis at extremes of age for this condition, and absence of prior asbestos exposure. In Case 1, a 20–25‐year‐old male with pericardial MM presented for left arm shaking, with brain MRI at this time revealing 14 total lesions, 85.7% of which had vasogenic edema. He underwent whole‐brain radiotherapy (WBRT), passing away 21.25 months following initial diagnosis. In Case 2, an 85–90‐year‐old male reported expressive aphasia and was found to have a large hemorrhagic frontotemporal lesion. He was subsequently diagnosed with pleural MM and received stereotactic radiosurgery (SRS) for management of BM, with a favorable treatment response on follow‐up imaging. He succumbed to systemic progression 6 months after diagnosis of BM. Next‐generation sequencing identified a missense mutation in *RAD51C* in Case 1, and *NF2* splice‐site and *TP53* frameshift mutations in Case 2.

**Conclusion:**

To our knowledge, this represents the first reported genetic profiling of MM with BM. The *TP53* frameshift mutation is unusual for MM, and its potential association with rapid disease progression warrants further investigation. Given the aggressive nature of MM, SRS may be preferable to WBRT due to its shorter treatment time and ease of combination with systemic regimens.

## 1 Introduction

Malignant mesothelioma (MM) is a rare, aggressive malignancy derived from mesothelial cells of the pleura, peritoneum, or pericardium [1]. MM represents only 0.2% of new cancer cases internationally [2], and is distinctive for its close association with asbestos [3]. Specifically, 70%–90% of pleural MM cases are considered attributable to asbestos exposure [4]. The prognosis for MM remains poor, with a median overall survival (OS) of 10.3 months from initial diagnosis [5]. In addition, the median age at diagnosis of MM is 70 years, with a significant male predominance [6]. While patient presentation is highly variable, adding to the diagnostic challenge of MM, the most common initial symptoms are chest pain and dyspnea [7].

Brain metastasis (BM) is an uncommon complication of MM, observed in only 2.7% of cases [8]. Notably, most published data concerning MM with BM were obtained from autopsy studies, with relatively few reports of the clinical presentation of this condition [8, 9, 10]. In addition, there are no prior reports of the genetic characteristics of MM with brain involvement. Significantly, recent studies suggest that pleural MM primary tumors have a low protein‐coding mutation rate by comparison with other solid cancers [11, 12]. Furthermore, pleural MM appears to be comprised of four molecular subgroups, with significant differences in OS [12]. Accordingly, biomarker‐based treatment stratification is beginning to be explored for pleural MM patients, with a recent clinical trial targeting disease negative for the tumor suppressor p16ink4A [13]. However, genetic characterization of MM remains lacking, particularly in atypical presentations, including central nervous system (CNS) metastasis, non‐asbestos‐related disease, and disease of pericardial origin.

Here, we present two unique cases that exhibited atypical presentations of MM with BM. Both patients were diagnosed at a highly unusual age for MM, exhibited neurological symptoms, and were found to have significant intracranial disease burden. In addition, neither patient had any prior history of asbestos exposure. We summarize immunohistochemical and next‐generation sequencing (NGS) results for both patients, which represent the first reported genetic profiling of MM with BM. Furthermore, we report the treatment course and outcomes for aggressive presentation of this disease. In both cases, written consent was obtained from the patient prior to initiation of treatment.

## 2 Cases

### 2.1 Case 1

**FIGURE 1:**
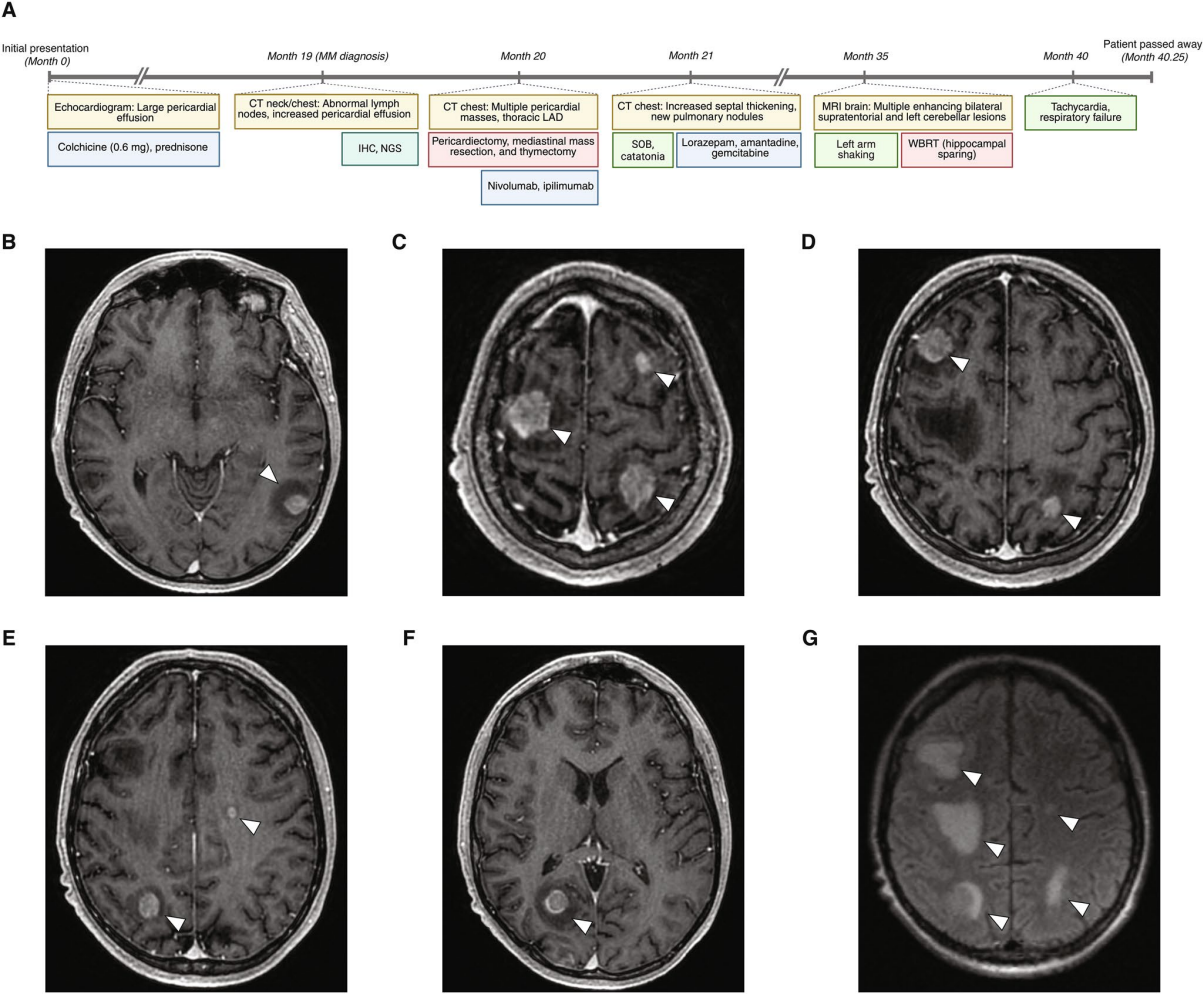
Summary of diagnosis, treatment, and follow‐up for Case 1. (A) Timeline of patient history of pericardial malignant mesothelioma with brain metastasis. IHC, immunohistochemistry; LAD, lymphadenopathy; MM, malignant mesothelioma; NGS, next‐generation sequencing; SOB, shortness of breath; WBRT, whole‐brain radiotherapy. (B–F) T1‐weighted post‐contrast axial MRI showing large intracranial tumor burden prior to WBRT, with lesions indicated by white arrowheads. Large lesions are visible in the right frontal lobe (2.3 × 2.2 cm), left postcentral gyrus (1.7 × 1.9 cm), right occipital lobe (1.4 × 1.3 cm), and left frontal lobe (0.4 × 0.5 cm). (G) Axial T2‐weighted FLAIR image demonstrating extensive perilesional edema prior to WBRT.

A 20–25‐year‐old male initially presented to an outside hospital for cough and dyspnea, leading to the identification of a large pericardial effusion on echocardiogram (Figure 1A). He was treated with a 3‐month course of colchicine (0.6 mg daily) and prednisone, given his normal left ventricular size and systolic function. Approximately 16 months following completion of treatment, he presented to Stanford University Medical Center for worsening lymphadenopathy and neck swelling. Neck CT showed abnormal lymph nodes along the left neck, including an enlarged left jugulodigastric node measuring 1.3 cm in diameter. Additionally, chest CT revealed increased mass‐like pericardial effusion, with subcutaneous soft tissue edema extending through the chest wall to the level of the trachea. Biopsy of the right paratracheal (4R) lymph node and immunophenotypic profiling showed the malignant cells to be WT1‐positive, P40‐negative, CK5/6‐positive, D2‐40‐positive, BerEP4‐negative, calretinin‐positive, and claudin‐4‐negative, with BAP1 retained. Collectively, this supported a diagnosis of epithelioid‐type MM of pericardial origin. Of note, there was a family history of early‐onset cancer, with his maternal aunt diagnosed with breast cancer and acute myeloid leukemia at ages 33 and 35, respectively. However, there were no known prior cases of MM within his family. In addition, NGS of a formalin‐fixed tumor biopsy tissue specimen identified a mutation in *RAD51C* [*c.492 T>G* (p.F164L)] (Tables 1 and S1). As paired germline testing was not performed, the somatic or germline origin of this variant could not be determined. Furthermore, the pathogenicity and functional relevance of this variant remain unknown [14, 15].

**TABLE 1:** Genetic characteristics of malignant mesothelioma with brain metastasis.

Case no.	Gene name	Alteration	VAF (%)	Genomic description	Transcript
1	*RAD51C*	*c.492 T>G* (p.F164L)	NA	chr17:g.58696746	NM_058216.3
2	*NF2*	*c.1731_1737del+7* (Splice‐site)	23	chr22:g.30077584	NM_000268.3
	*TP53*	*c.295_296del* (Frameshift)	27	chr17:g.7579391 _7579392del	NM_000546.5

Abbreviations: NA, not available; VAF, variant allele frequency.

One month later, he was admitted for shortness of breath. This was relieved by bilateral thoracentesis, with the pleural fluid found to contain malignant mesothelial cells. Chest CT at this time showed multiple pericardial masses nearly encasing the heart, thoracic and retrocrural lymphadenopathy, and interlobular septal thickening involving the right upper and right middle lobes. This septal thickening was believed to represent lymphangitic carcinomatosis. Furthermore, an interval increase in extensive filling defects throughout the venous system was noted. He subsequently underwent pericardiectomy, with resection of a mediastinal mass as well as thymectomy. Postoperative nivolumab and ipilimumab were administered.

At 2‐month follow‐up, chest CT showed increased diffuse nodular interlobular septal thickening, with greater involvement of the right compared with the left lung. In addition, new and increasing pulmonary nodules were noted, concerning for progression of disease. He was admitted to the emergency department 3 weeks later for shortness of breath. His course was complicated by catatonia, which was treated with lorazepam and amantadine. In addition, he was started on gemcitabine (1000 mg/m^2^).

Six months later, at 19 months following MM diagnosis, he presented with acute‐onset left arm shaking, prompting a concern for seizures. Brain MRI at this time showed multiple bilateral enhancing supratentorial lesions, with additional left cerebellar lesions (Figure 1B–F). This was consistent with metastatic disease, comprising 14 BM in total (Table 2). The largest lesions were located in the right frontal lobe (2.3 × 2.2 cm and 1.5 × 2.0 cm), left postcentral gyrus (1.7 × 1.9 cm), and right occipital lobe (1.4 × 1.3 cm). Vasogenic edema was observed surrounding 85.7% of lesions (Figure 1G). While the clinical presentation was highly suggestive of cortical seizure activity, this was not formally confirmed via electroencephalogram. The median diameter across all BM was 2.7 mm. Laterality was predominantly right‐sided (71.4%), with nine lesions located in the supratentorium and five in the cerebellum. There was no evidence of acute parenchymal hemorrhage. Collectively, this resulted in multifocal sulcal effacement without midline shift. These radiographic findings, combined with the neurologically symptomatic clinical presentation, suggested aggressive disease. At this time, he began levetiracetam (1 g BID) for seizure prophylaxis and dexamethasone (2 mg BID) for brain swelling.

**TABLE 2:** Summary of lesion‐level baseline characteristics for Case 1.

Factor	Number (%) or median (IQR)
Maximum diameter (mm)	2.7 (1.0, 8.1)
Laterality (right‐sided)	10 (71.4%)
Location	
Supratentorial	9 (64.3%)
Cerebellar	5 (35.7%)
Brainstem	0 (0%)
Edema	12 (85.7%)
Hemorrhage	0 (0%)

These BM were treated with whole‐brain radiotherapy (WBRT). Specifically, 30 Gy was delivered in 10 fractions, including six fractions with hippocampal avoidance. Three weeks following completion of WBRT, he presented to the emergency department of an outside hospital for tachycardia. He additionally reported persistent cough, voice changes, loss of taste, and significant anxiety since completion of WBRT. There was no evidence of respiratory infection, including negative testing for SARS‐CoV‐2. Additional work‐up revealed mild cardiomyopathy, thought to be related to tumor burden, with an ejection fraction of 50%. It was unknown to what extent intracranial disease may have contributed to cardiac and neuropsychiatric symptoms. He was admitted to the intensive care unit, where he was intubated for respiratory failure. He passed away shortly thereafter, 21.25 months following his initial diagnosis of pericardial MM.

### 2.2 Case 2

**FIGURE 2:**
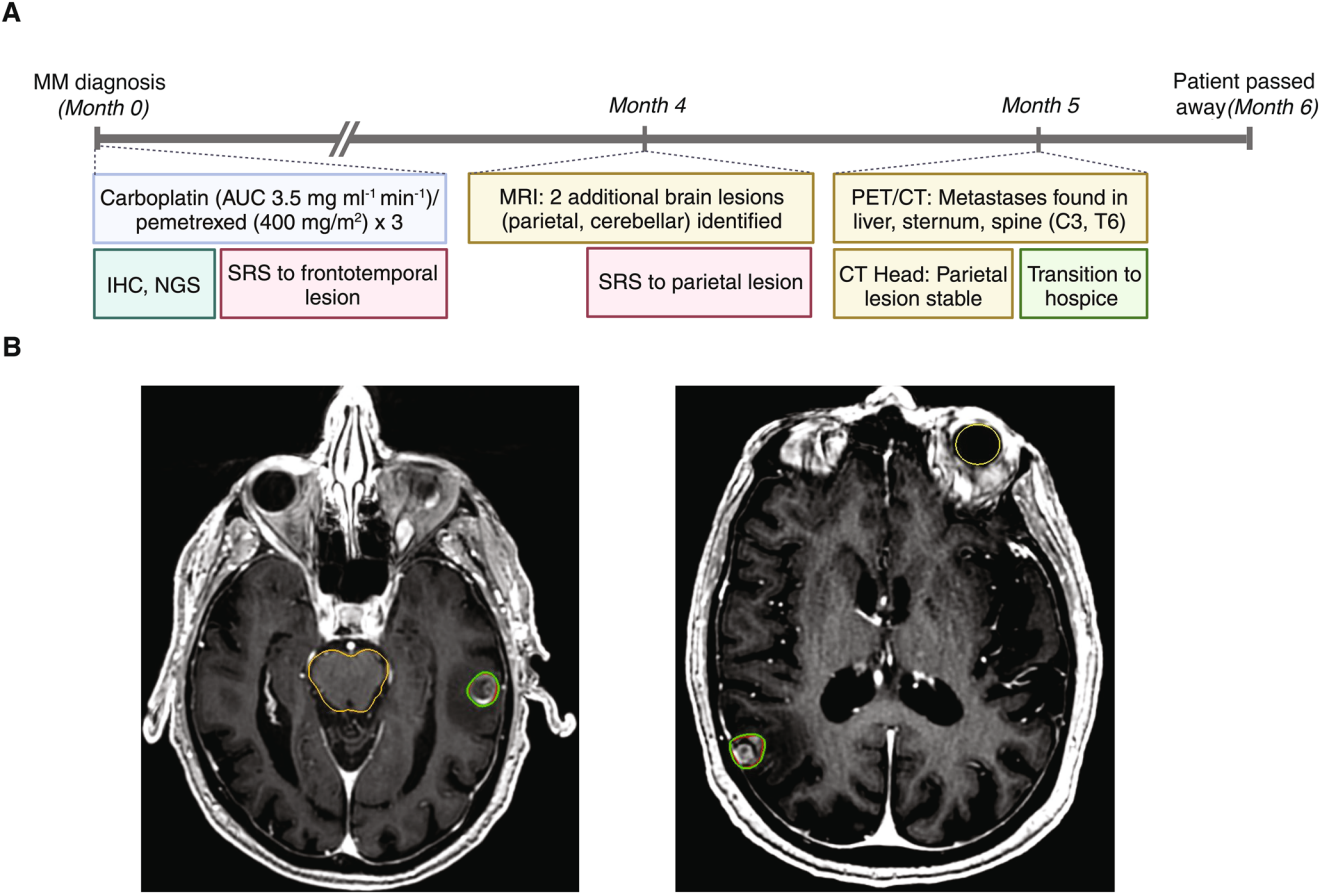
Summary of diagnosis, treatment, and follow‐up for Case 2. (A) Timeline of patient history of pleural malignant mesothelioma with brain metastasis. IHC, immunohistochemistry; MM, malignant mesothelioma; NGS, next‐generation sequencing; SRS, stereotactic radiosurgery. (B) CyberKnife radiosurgery plans on axial MRI. Treatment planning for the left temporal and right parietal lesions is shown on the left and right, respectively. Although a cerebellar lesion was identified concurrently with the parietal metastasis, it was managed via surveillance due to its radiographic stability. Red line: Planning target volume; Green line: Isodose line; Yellow line: Organs at risk.

An 85–90‐year‐old, right‐handed male with no prior history of neurological symptoms presented following an episode of expressive aphasia (Figure 2A). He had a previous diagnosis of nodular basal cell carcinoma of the right cheek, for which he underwent Mohs surgery 7 years prior. At the time of current presentation, brain MRI revealed a 1.5‐cm hemorrhagic lesion in the left frontotemporal lobe (Table 3). Further evaluation, including chest imaging and supraclavicular lymph node biopsy, confirmed pleural MM in the right lower lung. There was evidence of extensive involvement of the pleura in malignancy, as well as a single bone metastasis in the sternum. Lymph node metastases were additionally found in the right supraclavicular, mediastinal, and cardiophrenic regions, as well as a subdiaphragmatic lesion in the right celiac region. Lymph node biopsy was consistent with mesothelial origin, with strong and diffuse staining of CK5/6, calretinin, and WT1, as well as patchy D2‐40 expression. This was further supported by the absence of BerEP4, MOC‐31, TTF‐1, and Napsin‐A. In addition, PD‐L1 expression was found to be low (Tumor Proportion Score: 40%). Notably, the patient reported no history of occupational or environmental exposure to asbestos.

NGS identified a pathogenic splice‐site mutation in *NF2* [*c.1731_1737del+7*, variant allele frequency (VAF): 23%] and a *TP53* frameshift mutation (*c.295_296del*, VAF: 27%) (Table 1). The latter was predicted to lead to complete loss of normal TP53 protein function. Fluorescence in situ hybridization was negative for *ALK* and *ROS1* rearrangements. Furthermore, testing was negative for mutations in exons 18, 19, 20, and 21 of the *EGFR* gene.

Carboplatin (AUC 3.5 mg/mL/min) and pemetrexed (400 mg/m^2^) were initiated for systemic disease, with three total cycles administered. He additionally underwent stereotactic radiosurgery (SRS) to his frontotemporal BM, receiving 22 Gy in a single dose (Figure 2B). At 3‐month follow‐up, imaging showed a 16% volume reduction in the treated lesion. However, he was found to have two additional BM in the right parietal lobe and right inferior cerebellum. The decision was made to pursue additional SRS for treatment of the parietal BM, while the cerebellar lesion was monitored via surveillance imaging as it was considered more radiographically stable. He received a second treatment of SRS to the parietal lesion (maximum diameter: 15 mm, volume: 1.12 cm^3^) with a dose of 20 Gy delivered in one fraction. One month following SRS, head CT showed the treated parietal lesion to be stable. At this time, skull‐to‐thigh PET/CT identified new metastases in the liver, sternum, and spine (C3 and T6). The patient elected to transition to hospice care and passed away 1 month later, at 6 months following diagnosis of BM.

**TABLE 3:** Lesion‐level baseline characteristics and treatment details for Case 2.

Lesion no.	Location	Volume (cm^3^)	Maximum diameter (mm)	Prior resection	Treatment intent	Prescribed dose (Gy)	Isodose line (%)	No. of fractions	Maximum diameter at last follow‐up (mm)	Radiological response at last follow‐up
1	Left temporal	1.14	15	No	Definitive	22	75	1	13	Stable
2	Right parietal	1.12	15	No	Definitive	20	73	1	15	Stable

## 3 Discussion

Genomic characterization of MM has recently expanded and may soon play a role in prognosis. While the rarity of mesothelioma has historically posed an obstacle to large‐scale molecular analysis, multi‐institutional cohorts have provided significant insights. For example, such efforts have led to the establishment of genetic clusters of disease correlated with traditional histopathological classification [16, 17]. Importantly, only 10% of interpatient variability in mesothelioma is reported to be explainable by histopathology alone [16]. This underscores the need for further genetic characterization of this disease and its manifestations. Nevertheless, existing MM datasets are not annotated for BM, with no genetic profiles for MM with CNS involvement currently available. Therefore, the present study represents a meaningful contribution to the literature, which may inform our understanding of MM with this metastatic pattern.

The *TP53* mutation in Case 2 is significant, as *TP53* frameshifts are rarely seen in primary mesothelioma, suggesting a potential risk factor for CNS metastasis. While *TP53* is the most commonly mutated gene in human cancers, truncating mutations are much less frequent than missense mutations in tumors [18]. *TP53* inactivating mutations are rarer in MM, found in 5%–10% of tumors [17, 19, 20]. Accordingly, the frameshift variant identified in the present study is a true loss‐of‐function mutation, suggesting it may contribute to the unusual disease phenotype observed in our patient. An important limitation of our study is the reliance on VAF, whereas copy number variant (CNV) analysis would provide more robust evidence supporting loss of normal TP53 protein function. Future studies exploring TP53 in MM may use CNV to more comprehensively evaluate its role in disease progression.

The association between *TP53* mutations and CNS involvement in our cohort aligns with other solid malignancies, where such alterations may mark an aggressive phenotype predisposed to metastasis. Recent large‐scale genomic analyses identified *TP53* alterations as significant predictors of BM in breast cancer, showing a 1.91‐fold enrichment in HR^+^/HER2^−^ disease [21]. This link is supported by whole‐genome sequencing of basal‐like breast cancer, which confirmed the stable propagation of a *TP53* mutation from a primary tumor to a cerebellar metastasis [22], and observations of a high frequency of complex *TP53* mutations within breast cancer BM [23]. Similarly, comprehensive genomic profiling of over 3000 non‐small‐cell lung cancer (NSCLC) patients revealed that *TP53* alterations were significantly enriched in NSCLC BM compared with primary tumor samples [24]. In a separate study, *TP53* mutations in primary NSCLC were identified as significant predictors for subsequent development of BM, particularly when occurring alongside DNA damage repair pathway alterations [25]. Mechanistically, loss of wild‐type *TP53* may promote metastasis by removing transcriptional barriers to the epithelial–mesenchymal transition and anoikis while creating a permissive environment for genomic instability [26]. Paradoxically, specific *TP53* hotspot mutations have also been suggested to have gain‐of‐function effects which enhance metastatic potential [26, 27]. While this suggests that *TP53* dysfunction may enhance the likelihood of CNS involvement by driving general metastatic potential, the clinical evidence remains largely correlative. It has yet to be determined whether *TP53* alterations act as specific molecular drivers of brain tropism or if they simply represent a marker of a more aggressive, high‐grade phenotype with a generalized capacity for dissemination. Further mechanistic studies are therefore required to investigate these two possibilities.

The clinical relevance of the *RAD51C* p.F164L variant remains difficult to characterize, as it is considered a variant of uncertain significance. Although identified in some germline cohorts of familial breast and ovarian cancer, it has also been recorded in population databases such as gnomAD among healthy individuals, which complicates its classification as a high penetrance pathogenic allele [14]. Recent functional studies indicate that while the p.F164L substitution disrupts the physical interaction between RAD51C and its partner RAD51D, it does not appear to significantly impair overall homologous recombination repair capacity or increase sensitivity to platinum‐based therapies [15]. The clinical significance of the five paralogs of the RAD51 recombinase (XRCC2, XRCC3, RAD51B, RAD51C, and RAD51D) remains an area of active investigation [28], there is currently insufficient evidence to conclude whether the specific genetic background or the primary site was the dominant driver of CNS dissemination in Case 1.

Notably, Case 1 describes the treatment course of BM from pericardial MM, an exceedingly rare and aggressive malignancy. Pericardial MM comprises 0.7% of mesothelioma cases [29], with an incidence of 0.0022% [30]. Prior reports of BM from pericardial MM remain exceedingly rare, with limited documentation currently available in the English literature [31, 32]. Consequently, genetic and immunohistochemical characterization of pericardial MM is also lacking. A recent pathology study of three cases of pericardial MM found there to be significant overlap in morphology, genetic profile, and molecular markers with pleural MM, including inactivation of the tumor suppressors *p16*, *NF2*, and *TP53* in one mesothelioma case, each [33]. This is generally consistent with our findings in the present study, wherein both Cases 1 and 2 had similar immunohistochemical profiles. Importantly, to date only six cases of pericardial MM across three studies have been analyzed with NGS [33, 34, 35], such that our results represent a meaningful addition to the literature. In addition, the etiologic factors of pericardial MM remain largely unknown, with further investigation needed to elucidate the mechanisms underlying this condition. There is conflicting evidence regarding the association between asbestos exposure and pericardial MM, with detection of asbestos fibers in the lungs of some patients but absent in others [33]. The histology observed in our case is consistent with prior immunohistochemical analyses of pericardial MM, given that the majority of published cases were reported to be epithelioid or biphasic [34, 36]. Overall, a stronger understanding of the molecular drivers of this highly rare condition may facilitate future diagnostic and treatment efforts.

The clinical presentations observed in both Cases 1 and 2 are highly unusual for MM, suggesting an aggressive disease course with rapid growth of intracranial lesions. Arm shaking and concern for seizure led to the discovery of BM in Case 1. Similarly, the chief complaint in Case 2 was expressive aphasia, with a subsequent diagnosis of MM. Manifestation of neurological signs of MM, particularly in the absence of extracranial symptoms, is extraordinarily uncommon [37]. In addition, both patients initially presented at a highly atypical age for MM, 20–25 and 85–90 years old, which significantly differs from the median age of diagnosis for this condition [6]. Furthermore, there appeared to be a rapid increase in intracranial disease burden in both cases. Case 1 presented with a large number of widely distributed BM, including several notably large lesions, while Case 2 developed additional lesions shortly after completion of initial SRS. Additionally, Case 1 quickly deteriorated after WBRT, precluding follow‐up for his BM. Finally, neither patient had a prior history of asbestos exposure, either occupational or personal.

Growing evidence suggests SRS may be superior to WBRT for BM in the setting of a large number of intracranial lesions [38, 39, 40]. For example, patients with up to 15 BM have been found to benefit from SRS [38]. In particular, this suggests that SRS may be appropriate as a first‐line treatment for select patients with aggressive disease, including for a high number of BM or large individual lesions. Notably, for lesions > 3 cm, dose fractionation is preferred to single‐fraction SRS in order to minimize risk of radiation necrosis [41, 42]. The OS of 6 months in Case 2 is relatively favorable, with only 13 reported cases of mesothelioma BM surviving for ≥ 6 months [43]. These findings support the efficacy of SRS for this rare condition, although further research may be needed to improve patient stratification. This is consistent with prior documentation of SRS for MM BM, which have reported posttreatment survival ranging from 1 to 7 months [37, 43, 44, 45, 46, 47]. In Case 1 of the current study, however, it was notably difficult to determine the degree of benefit obtained from WBRT, given the short available follow‐up.

More broadly, identifying the subset of MM patients for which SRS is preferable to WBRT may involve multimodal investigation, including genetic testing. Genomic profiling is receiving increasing attention as a means of predicting radiation response, particularly for cases in which clinical predictors remain limited [40, 48, 49]. Given the high heterogeneity of MM and the therapeutic challenges this presents, molecularly informed approaches may be particularly valuable.

## 4 Conclusion

We present the first report of genetic profiling of MM with BM. Both patients exhibited neurological symptoms, presented at unusual ages for MM, and had no prior history of asbestos exposure. Notably, genetic analysis revealed a *TP53* frameshift mutation in one patient, a severe mutation for this disease which warrants further investigation for a potential association with increased metastatic spread, including to the CNS. In addition, we summarize the treatment course for extensive BM from pericardial MM, an exceedingly rare primary tumor type, which resulted in a relatively favorable OS for this condition. Despite receiving chemotherapy and radiation, both patients experienced systemic progression, ultimately leading them to succumb to disease. These cases underscore the need for further genetic characterization of MM, particularly in atypical presentations, to enhance prognostic and treatment strategies. In addition, early and aggressive treatment may be warranted for MM with intracranial involvement, given the potential for rapid progression observed in this rare condition.

## Author Contributions

**Paul M. Harary:** conceptualization, investigation, writing – original draft, visualization, writing – review and editing, formal analysis, data curation. **Yusuke S. Hori:** conceptualization, writing – review and editing, supervision, formal analysis. **Ruchit Jain:** writing – review and editing. **Ahed H. Kattaa:** writing – review and editing. **Melanie Hayden Gephart:** writing – review and editing. **Michael F. Gensheimer:** writing – review and editing. **Scott G. Soltys:** writing – review and editing. **David J. Park:** conceptualization, writing – review and editing, resources, supervision. **Steven D. Chang:** conceptualization, writing – review and editing, resources, supervision.

## Funding

The authors have nothing to report.

## Ethics Statement

This study was approved by the Stanford University Institutional Review Board (IRB #3910). The IRB granted a waiver of written informed consent for the publication of patient data due to the retrospective design and the use of fully de‐identified information.

## Consent

Written patient consent for treatment was obtained prior to initiation of procedures. The authors made multiple attempts to contact the next of kin for publication of case details but were unable to obtain a response. No patient identifying information or identifying images are included in this manuscript.

## Conflicts of Interest

The authors declare no conflicts of interest.

## Supporting information


**Table S1:** Full panel of tested genes.

## Data Availability

The data that support the findings of this study are available on request from the corresponding author. The data are not publicly available due to privacy or ethical restrictions.
