# Supplementary material for: Genetic and Immunohistochemical Profiling of Malignant Mesothelioma With Brain Metastasis: A Report of Two Cases
Source: Cancer Rep (Hoboken). 2026 May 8;9(5):e70571. doi: 10.1002/cnr2.70571 (PMC13154776; doi:10.1002/cnr2.70571)
Supplement: Supplementary file 1 — Table S1: Full panel of tested genes. [file CNR2-9-e70571-s001.docx]

**Table S1.** Full Panel of Tested Genes

| *ABL1* | *CCND2* | *DPH3* | *FGFR3* | *IKZF3* | *MYBL1* | *POLE* | *SMARCA4* |
| --- | --- | --- | --- | --- | --- | --- | --- |
| *AKT1* | *CCND3* | *DROSHA* | *FH* | *JAK2* | *MYC* | *POLR2A* | *SMARCB1* |
| *AKT2* | *CCNE1* | *EGFR* | *FLCN* | *JAK3* | *MYCL* | *POT1* | *SMARCE1* |
| *AKT3* | *CD274* | *EIF1AX* | *FLT3* | *KDM6A* | *MYCN* | *PPP2R1A* | *SMO* |
| *ALK* | *CDH1* | *ELOC* | *FOXA1* | *KDR* | *MYD88* | *PRKCA* | *SOX2* |
| *APC* | *CDK12* | *EP300* | *FOXL2* | *KEAP1* | *NF1* | *PTCH1* | *SPOP* |
| *AR* | *CDK4* | *EPHA2* | *FOXO1* | *KIT* | *NF2* | *PTEN* | *SRC* |
| *ARAF* | *CDK6* | *EPHA3* | *FOXR2* | *KLF4* | *NFE2L2* | *PTPN11* | *SRSF2* |
| *ARID1A* | *CDKN1B* | *ERBB2* | *FUBP1* | *KRAS* | *NKX2-1* | *RAC1* | *STAG2* |
| *ATM* | *CDKN2A* | *ERBB3* | *GATA3* | *LRP1B* | *NOTCH1* | *RAF1* | *STK11* |
| *ATR* | *CDKN2B* | *ERBB4* | *GLI1* | *LZTR1* | *NOTCH3* | *RB1* | *SUFU* |
| *ATRX* | *CDKN2C* | *ERCC1* | *GNA11* | *MAP2K1* | *NRAS* | *RELA* | *TACC3* |
| *AURKA* | *CHEK1* | *ERCC2* | *GNAQ* | *MAP2K2* | *NTRK1* | *RET* | *TERT* |
| *AXIN1* | *CHEK2* | *ERRFI1* | *GNAS* | *MCL1* | *NTRK2* | *RHEB* | *TP53* |
| *BAP1* | *CIC* | *ESR1* | *H3-3A* | *MDM2* | *NTRK3* | *RHOA* | *TP63* |
| *BCL2* | *CREBBP* | *EWSR1* | *H3-3B* | *MDM4* | *PALB2* | *RIT1* | *TRAF7* |
| *BCOR* | *CTNNB1* | *EZH2* | *H3C2* | *MED12* | *PCBP1* | *RNF43* | *TSC1* |
| *BCR* | *CUL3* | *FANCA* | *HGF* | *MEN1* | *PDGFRA* | *ROS1* | *TSC2* |
| *BRAF* | *CXCR4* | *FANCF* | *HNF1A* | *MET* | *PDGFRB* | *SDHB* | *U2AF1* |
| *BRCA1* | *CYLD* | *FANCG* | *HOXB13* | *MLH1* | *PHOX2B* | *SDHC* | *VEGFA* |
| *BRCA2* | *CYSLTR2* | *FBXW7* | *HRAS* | *MPL* | *PIK3CA* | *SDHD* | *VHL* |
| *BRD4* | *DDR2* | *FGF3* | *IDH1* | *MSH2* | *PIK3R1* | *SETBP1* | *WT1* |
| *BTK* | *DDX3X* | *FGF4* | *IDH2* | *MSH6* | *PLEKHS1* | *SETD2* | *YAP1* |
| *CASP8* | *DICER1* | *FGFR1* | *IGF1R* | *MTOR* | *PMS2* | *SF3B1* | *ZMYM3* |
| *CCND1* | *DNMT3A* | *FGFR2* | *IKBKE* | *MYB* | *POLD1* | *SMAD4* |  |

**Additional intronic coverage for fusion detection**

| *ALK* | *EGFR* | *FGFR1* | *FOS* | *MAP3K8* | *NTRK2* | *RET* | *TFEB* |
| --- | --- | --- | --- | --- | --- | --- | --- |
| *BRAF* | *ETV6* | *FGFR2* | *FUS* | *MYB* | *RAF1* | *ROS1* | *YAP1* |
| *CD74* | *EWSR1* | *FGFR3* | *MET* | *NTRK1* | *RELA* | *TFE3* |  |
